# Neuron-derived transthyretin modulates astrocytic glycolysis in hormone-independent manner

**DOI:** 10.18632/oncotarget.22542

**Published:** 2017-11-20

**Authors:** Alina Zawiślak, Piotr Jakimowicz, James A. McCubrey, Dariusz Rakus

**Affiliations:** ^1^ Department of Physiology and Molecular Neurobiology, Faculty of Biological Sciences, University of Wroclaw, Wroclaw, Poland; ^2^ Department of Protein Engineering, Faculty of Biotechnology, University of Wroclaw, Wroclaw, Poland; ^3^ Department of Microbiology and Immunology, Brody School of Medicine at East Carolina University Greenville, Greenville, NC, USA

**Keywords:** astrocyte-neuron lactate shuttle, brain metabolism, cAMP/PKA signalling, GSK3, PI3K/AKT pathway

## Abstract

It has been shown that neurons alter the expression of astrocytic metabolic enzymes by secretion of until now unknown molecule(s) into extracellular fluid. Here, we present evidence that neuron-derived transthyretin (TTR) stimulates expression of glycolytic enzymes in astrocytes which is reflected by an increased synthesis of ATP. The action of TTR is restricted to regulatory enzymes of glycolysis: phosphofructokinase P (PFKP) and pyruvate kinase M1/M2 isoforms (PKM1/2). The regulation of PFK and PKM expression by TTR is presumably specific for brain tissue and is independent of the role of TTR as a carrier protein for thyroxine and retinol. TTR induced expression of PKM and PFK is mediated by the cAMP/PKA-dependent pathway and is antagonized by the PI3K/Akt pathway. Our results provide the first experimental evidence for action of TTR as a neuron-derived energy metabolism activator in astrocytes and describe the mechanisms of its action. The data presented here suggest that TTR is involved in a mechanism in which neurons stimulate degradation of glycogen-derived glucosyl units without significant modulation of glucose uptake by glial cells.

## INTRODUCTION

The brain is characterised by very high energy requirements and glucose is the main energetic substrate for this organ [1]. During the last two decades a growing body of evidence has accumulated supporting the hypothesis of the astrocyte-neuron lactate shuttle (ANLS) that postulates that the majority of glucose taken up by brain from the blood stream is utilized by astrocytes which convert it into lactate that is used to fulfill energy requirements of neurons [2, 3]. It has also been shown that glutamate released by active neurons stimulates glycolysis in astrocytes leading to the increased production of lactate [2].

The tight coupling between astrocytic metabolism and synaptic transmission suggests that these two cell types can act as a kind of syncytium, cross-regulating expression of proteins involved in energy metabolism and neurotransmitter synthesis. In accordance with this concept, our previous study has demonstrated that co-culturing neurons with astrocytes significantly affected the level of mRNA and protein abundance of the metabolic enzymes in both cell types. Moreover, we have provided evidence that neurons augment the expression of astrocytic enzymes both by direct physical contact of the cells and by release of, as yet unknown, molecule(s) into extracellular fluid [4]. In accordance with our results, it has been recently shown that neurons may regulate, via Notch signalling, the expression of hundreds of astrocytic genes, among them ones involved in ANLS, however, the changes were observed only at the transcriptome level [5].

In the present study, we show the results of studies focused on the identification of the regulatory factor(s) released from neurons into extracellular fluid and stimulating astrocyte energy metabolism. We demonstrate that neuron-released TTR stimulates the expression of glycolytic enzymes in astrocytes which results in an increased synthesis of ATP. Remarkably, the action of TTR is restricted to the regulators of glycolysis, phosphofructokinase P (PFKP) and pyruvate kinase M1/M2 isoforms (PKM1/2).

TTR is an evolutionary conserved [6, 7] carrier protein for thyroxine (T4) [8] and retinol [9]. In the brain, TTR is mainly synthetized by ependymocytes [10], but also by neurons [11] and its receptors are expressed, among others, on astrocytes [12]. The role of TTR in the biology of the nervous system has been described in several contexts. It has been shown to be involved in cognition [13, 14], behaviour [15], neuropeptide maturation [16] and nerve regeneration [17]. TTR has also been shown to play a protective role in Alzheimer’s disease [18, 19] and cerebral ischemia [20].

The results presented here reveal that TTR modulates glial energy metabolism independently of its role as a carrier protein. We also demonstrate that only the forms of TTR unsaturated by hormones (T4 and retinol) may stimulate the expression of PKM1/2. Moreover, the stimulation is mediated by the cAMP-dependent pathway and it is antagonized by the PI3K/AKT pathway.

To the best of our knowledge, this is the first report identifying the neuron-derived energy metabolism regulator of astrocytes and describing its mechanism of action.

## RESULTS

### Neuron-derived metabolic regulator(s) are heat unstable and with molecular mass between 30 and 100 kDa

**Figure 1:**
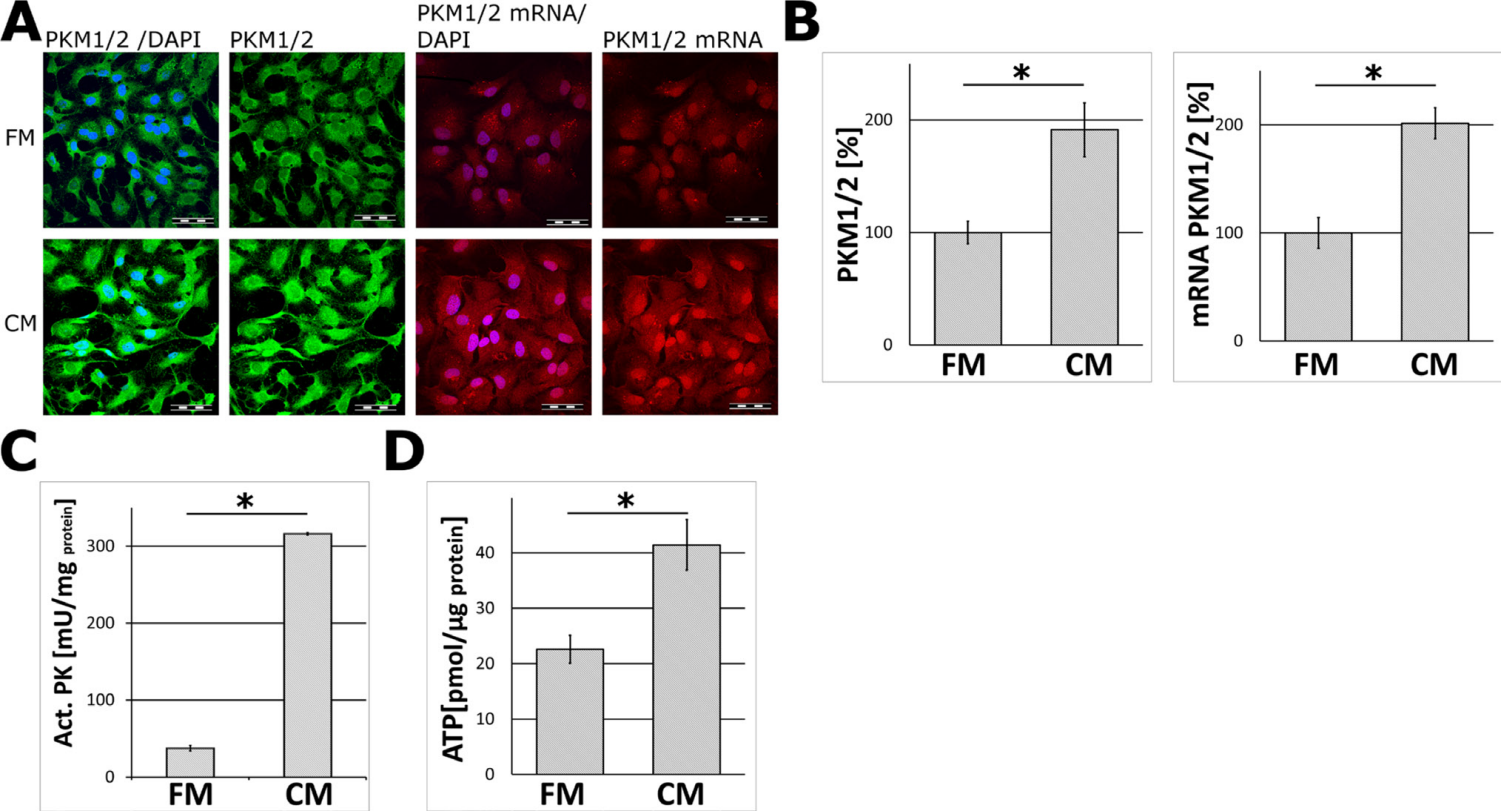
The effect of the neuron-conditioned medium (CM) on ATP synthesis and the levels of mRNA, protein abundance and the activity of pyruvate kinase (PKM1/2) in astrocytes (**A**) Subcellular localization of mRNA (right panel) and protein (left panel) of PKM1/2 in astrocytes cultured for 24 h or 48 h respectively in the neuron-conditioned medium (CM) or the neuronal fresh medium (FM). The nuclei were visualized with DAPI. Bar = 50 μm. (**B**–**D**) The effect of CM on: (B) the expression of the PKM1/2 protein (left chart) and mRNA (right chart), (C) the activity of PKM1/2, (D) ATP synthesis in astrocytes. The expression of the enzyme in cells cultured in the FM medium was assumed to be 100%. Each value represents the mean and S.D. of at least three individual experiments. Asterisks (^*^) indicate statistically significant differences (*P* < 0.001).

The incubation of astrocytes for 24 and 48 hours in the neuron-conditioned medium (CM) significantly elevated the level of mRNAs, protein abundance and activities for both isozymes of pyruvate kinase (PKM1/2) as compared to the cells cultured in the fresh neuronal medium (FM) (Figure 1A–1C). Immunofluorescence analysis revealed that treatment with the CM did not affect the morphology of the cells as compared to unstimulated (the FM-treated) astrocytes (Supplementary Figure 2).

To ensure that the observed changes did not result from substitution of a standard astrocytic culture medium (DMEM/FBS-based) with the neuronal one (neurobasal/ B27-based), we compared PKM1/2 expression in both the media and we did not find any differences in PKM1/2 expression (Supplementary Figure 3).

**Figure 2:**
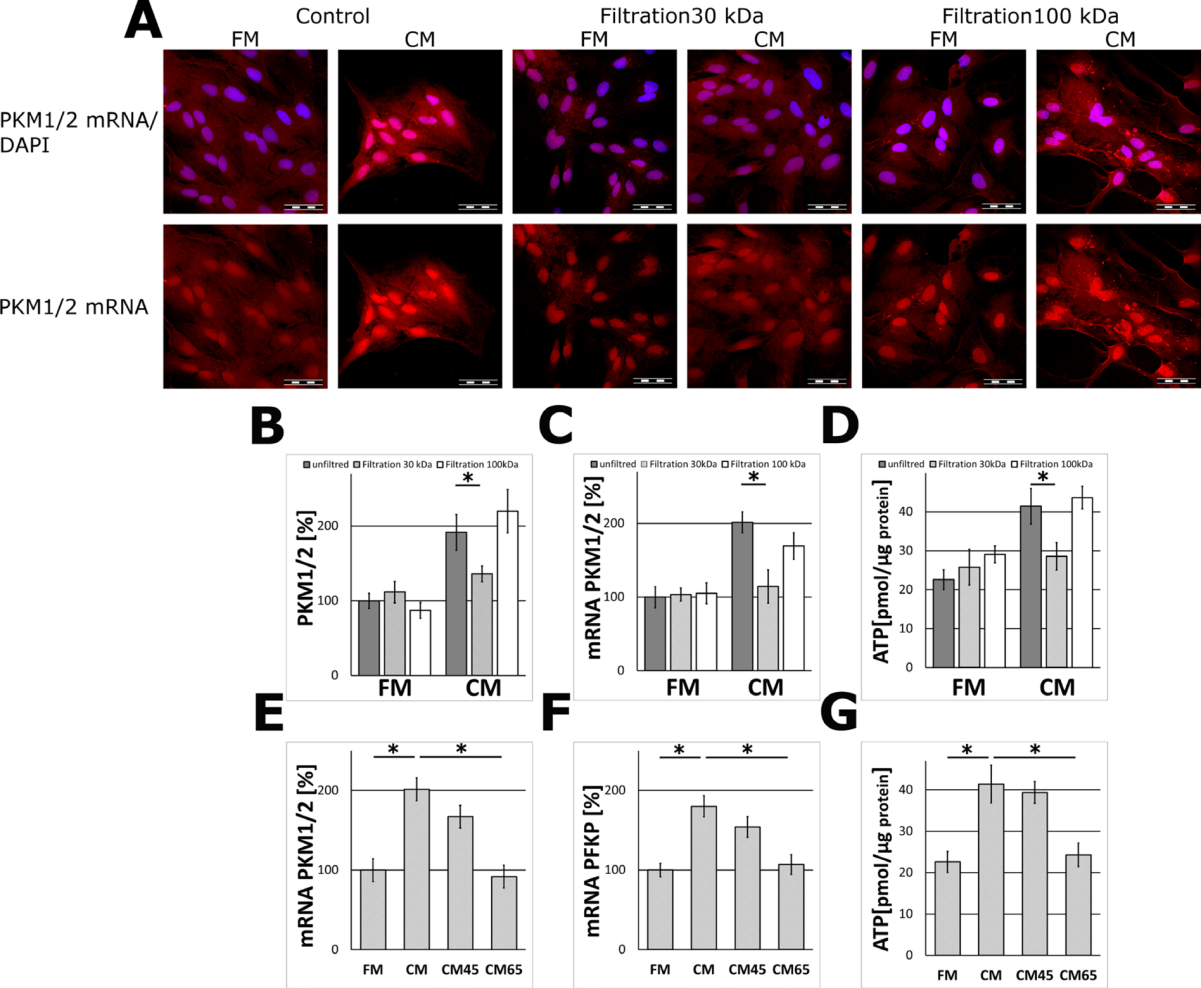
The effect of filtration (MWCO 30 and 100 kDa) and heat inactivation of the neuron-conditioned medium on PKM1/2 and PFKP expression in astrocytes (**A**) Subcellular localization of mRNA encoding PKM1/2 in astrocytes cultured for 24 h in the CM or the FM and the media fractions containing molecules of the mass below 30 kDa (“Filtration 30 kDa”) or below 100 kDa (“Filtration 100 kDa”). The nuclei were visualized with DAPI. Bar = 50 μm. (**B**–**D**) The effect of filtration (MWCO 30 and 100 kDa) of the media on the PKM1/2 protein (B) and mRNA (C) expression and ATP synthesis (D) in astrocytes. (**E**–**G**) The effect of heat inactivation of the CM (5 min at 45°C–“CM45” or at 65°C – “CM65”) on the level of mRNA for PKM1/2 (E) and PFKP (F) and ATP synthesis (G) in astrocytes. The expression of the enzyme in cells cultured in the FM medium was assumed to be 100%. Each value represents the mean and S.D. of at least three individual experiments. Asterisks (^*^) indicate statistically significant differences (*P <* 0.001).

Heat-treatment of the CM samples resulted in the loss of the ability of the medium to stimulate PKM1/2 and PFKP expression in astrocytes suggesting that the “neuron-derived effector” may be a protein or polypeptide (Figure 2E, 2F).

The results of molecular filtration studies demonstrated that fraction of the CM containing molecules with the mass in the range of 30–100 kDa was able to elevate PKM1/2 expression in the glial cells (Figure 2A–2C).

### Neuron-derived factor(s) stimulate ATP synthesis in astrocytes

Since pyruvate kinase is a regulatory enzyme of glycolysis, it might be expected that treatment of astrocytes with the CM impact the energy production in these cells. To address this issue, astrocytes were treated with the CM for 48 h and this increased about 2-fold the level of ATP as compared to cells incubated in the FM medium (Figure 1D). This effect was abolished after a heat-treatment of the CM (Figure 2G). A similar increase in the ATP level was observed after incubation of astrocytes with a fraction of the CM medium containing components of the mass below 100 kDa, but not those of the mass lower than 30 kDa (Figure 2D).

### Transthyretin is detected in neuron-conditioned medium

To determine which proteins are released into extracellular fluid by neurons, fractions of 30–100 kDa of the FM and the CM media were subjected to MS analysis. All recorded proteins are presented in Supplementary Table 1. Among the proteins detected in the CM medium, only transthyretin and enoyl-CoA hydratase domain-containing protein 3 (ECHDC3) were unique for this medium. The molecular mass of ECHDC3 is 33 kDa, and the mass of TTR tetramer (a physiological oligomeric state of the protein) is 55 kDa. However, ECHDC3 is an intracellular protein expressed in mitochondrion and its presence in the CM probably resulted from cells lysis, thus, TTR was assumed to be the neuron-derived metabolic regulator and used in further studies.

### Transthyretin is neuron-derived metabolic regulator

**Figure 3:**
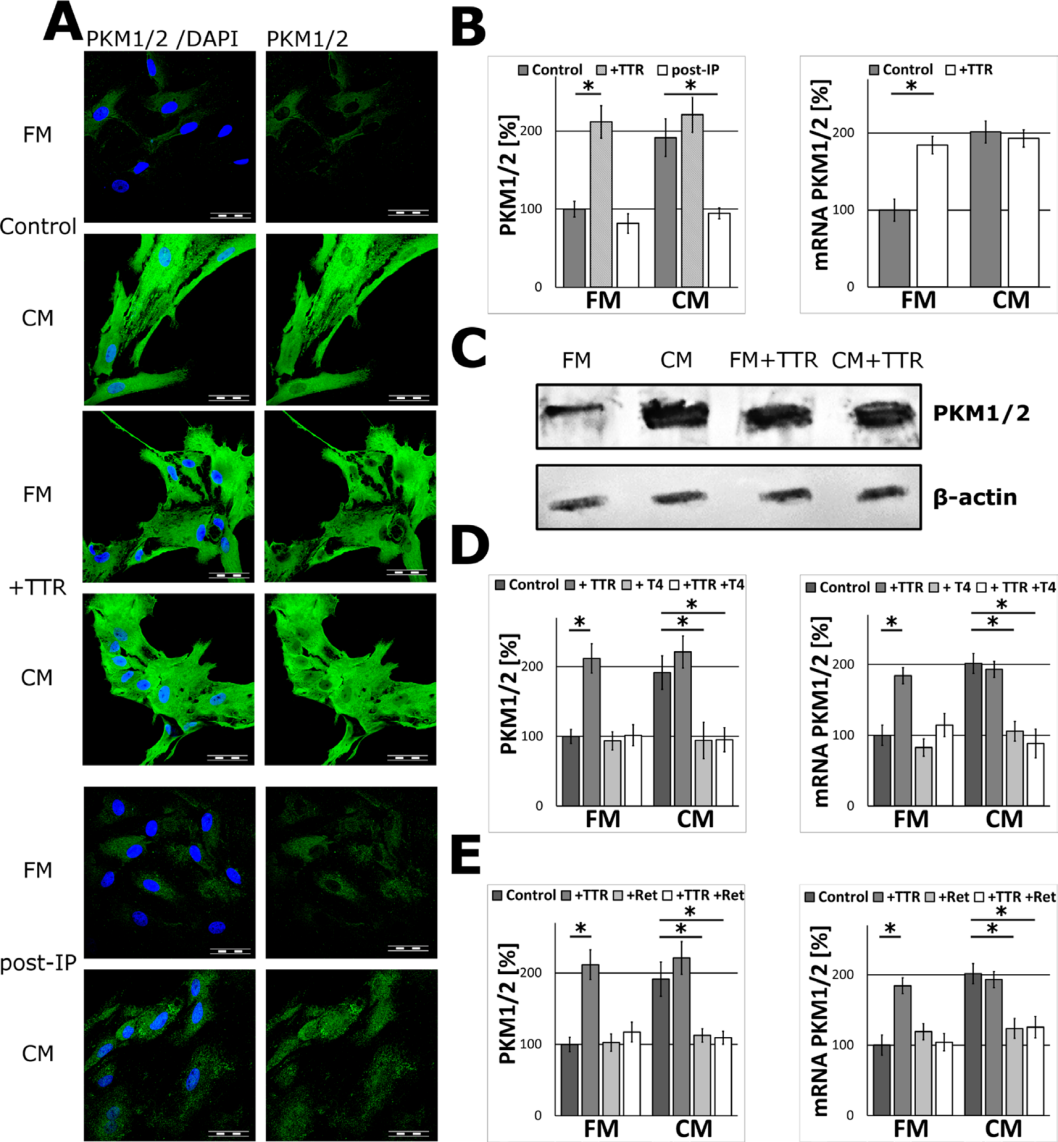
The effect of TTR on PKM1/2 expression in astrocytes (**A**–**B**) The effect of supplementation of the media with 36.4 nM TTR (+TTR) and depletion of TTR from the media (post-IP) on the subcellular localization (A) and expression of PKM1/2 protein (B; left chart) and mRNA (B; right chart) in astrocytes. The nuclei were visualized with DAPI. Bar = 50 μm. (**C**) Western blot analysis of PKM1/2 in extracts (15 μg of total protein per line) of astrocytes cultured for 48 h in the FM and the CM media supplemented with 36.4 nM TTR (+TTR); β-actin was used as a loading control. (**D**–**E**) The effect of saturation of TTR with 1 μM L-thyroxine, T4 (D) or 1 μM retinol, Ret (E) on the expression of the PKM1/2 mRNA (right chart) and protein (left chart) in astrocytes. The expression of the enzyme in cells cultured in the FM medium was assumed to be 100%. Each value represents the mean and S.D. of at least three individual experiments. Asterisks (^*^) indicate statistically significant differences (*P <* 0.001).

Human plasma TTR at physiological concentration (20 μg/ml, 36.4 nM) [21, 22] was added to the FM medium to test its ability to activate the expression of glycolytic enzymes in glial cells. The results of immunofluorescent study, FISH experiments and WB analysis revealed that TTR elevated the level of PKM1/2 in a similar manner to that observed for the CM-activated astrocytes. The supplementation of media with TTR did not affect the morphology of the cells (Supplementary Figure 2). The supplementation of the CM with TTR did not cause further increase in the expression of PKM1/2 (Figure 3A–3C).

To investigate if the stimulating effect of TTR was associated with its role as a carrier protein for T4 and retinol, the FM medium was supplemented with TTR-saturating concentrations (1 μM) of L-thyroxine or retinol. As a result, the stimulatory effects of TTR were abolished. Moreover, when T4 or retinol were added to the CM, the ability of such mixtures to stimulate expression of PKM1/2 was abrogated and the level of PKM1/2 was practically the same as the FM medium (Figure 3D, 3E). From this, it is evident that only the ligand-free TTR played a significant role of signalling molecule increasing PKM1/2 expression in astrocytes.

To further verify the role of TTR, the CM samples were deprived of the protein using immunoprecipitation. As it is shown in Figure 3A, 3B such treatment of the CM medium resulted in a significant abrogation of the CM ability to stimulate the expression of PKM1/2 in astrocytes both at mRNA and protein levels (Figure 3A, 3B).

**Figure 4:**
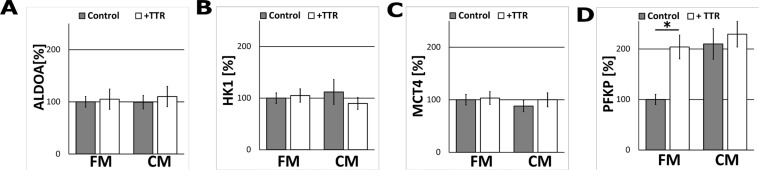
The effect of TTR and the CM on ALDOA, HK1, MCT4 and PFKP expression in astrocytes The measurement of ALDOA (**A**), HK1 (**B**), MCT4 (**C**), PFKP (**D**) protein-related immunofluorescence in astrocytes cultured for 48 h in the CM and in the FM supplemented with 36.4 nM TTR (+TTR). The expression of the enzyme in cells cultured in the FM medium was assumed to be 100%. Each value represents the mean and S.D. of at least three individual experiments. Asterisks (^*^) indicate statistically significant differences (*P <* 0.001).

In order to determine which other steps of glycolysis in astrocytes were modulated by TTR, the impact of the CM and TTR-treatment on the expression of hexokinase 1 (HK1, which catalyses the first step of glycolysis), aldolase A (ALDOA, which catalyses the central reaction of glycolysis), PFKP (the second regulatory enzyme of glycolysis) and monocarboxylate transporter 4 (MCT4, the astrocytic lactate transporter) were studied. Immunofluorescence analysis revealed that treatment with the CM nor TTR did not affect the expression of ALDOA and MCT4 as compared to unstimulated (the FM-treated) astrocytes (Figure 4A, 4C). At the same time, the expression of PFKP was affected in a similar manner as PKM1/2 (Figure 4D). Interestingly, the expression of HK1 remained unchanged (Figure 4B).

To examine the dynamism of TTR modulation, the expression of metabolic enzymes in the CM and TTR-treated cells was studied at different time points. TTR-induced changes in mRNA level for PFK and PKM were observed already after 30 minutes and they progressed in time (Supplementary Figure 5A). The changes in the proteins expression were much slower since they were observed only after 48 h (Supplementary Figure 5B).

**Figure 5:**
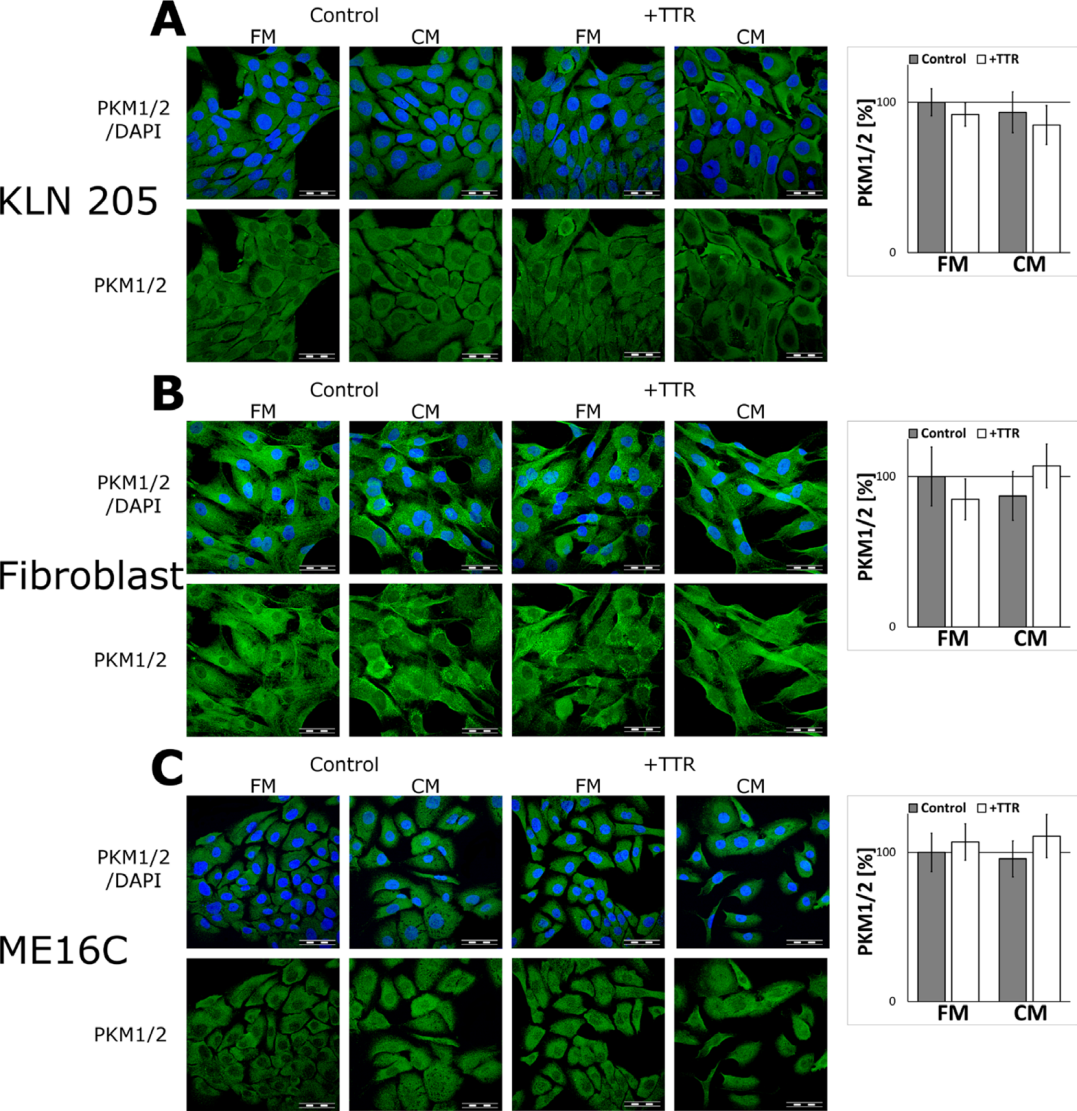
The effect of the CM and TTR on the expression of PKM1/2 in non-glial cells The cells were cultured for 48 h in the FM or the CM media supplemented with 36.4 nM TTR (+TTR). (**A**–**C**, left panels) Subcellular localisation of PKM1/2 in the KLN205 (A), human skin fibroblasts (B) and the ME16C cells (C). Right charts in each panel (A–C) show quantitation of PKM1/2-related fluorescence. The expression of the enzyme in cells cultured in the FM was assumed to be 100%. Each value represents the mean and S.D. of at least three individual experiments. The nuclei were visualized with DAPI. Bar = 50 μm.

To corroborate that TTR acts specifically to glial cells, the effect of the CM and TTR on the expression of PKM1/2 in non-glial cells was tested. The results of immunofluorescent studies revealed no changes in the expression of glycolytic enzymes in mouse lung cancer cell line (KLN205), human skin fibroblasts, nor human immortalized epithelial cells (ME16C) after stimulation with TTR (Figure 5).

### TTR stimulates PKM1/2 expression via the cAMP-dependent pathway and it is antagonized by the PI3K/AKT pathway

**Figure 6:**
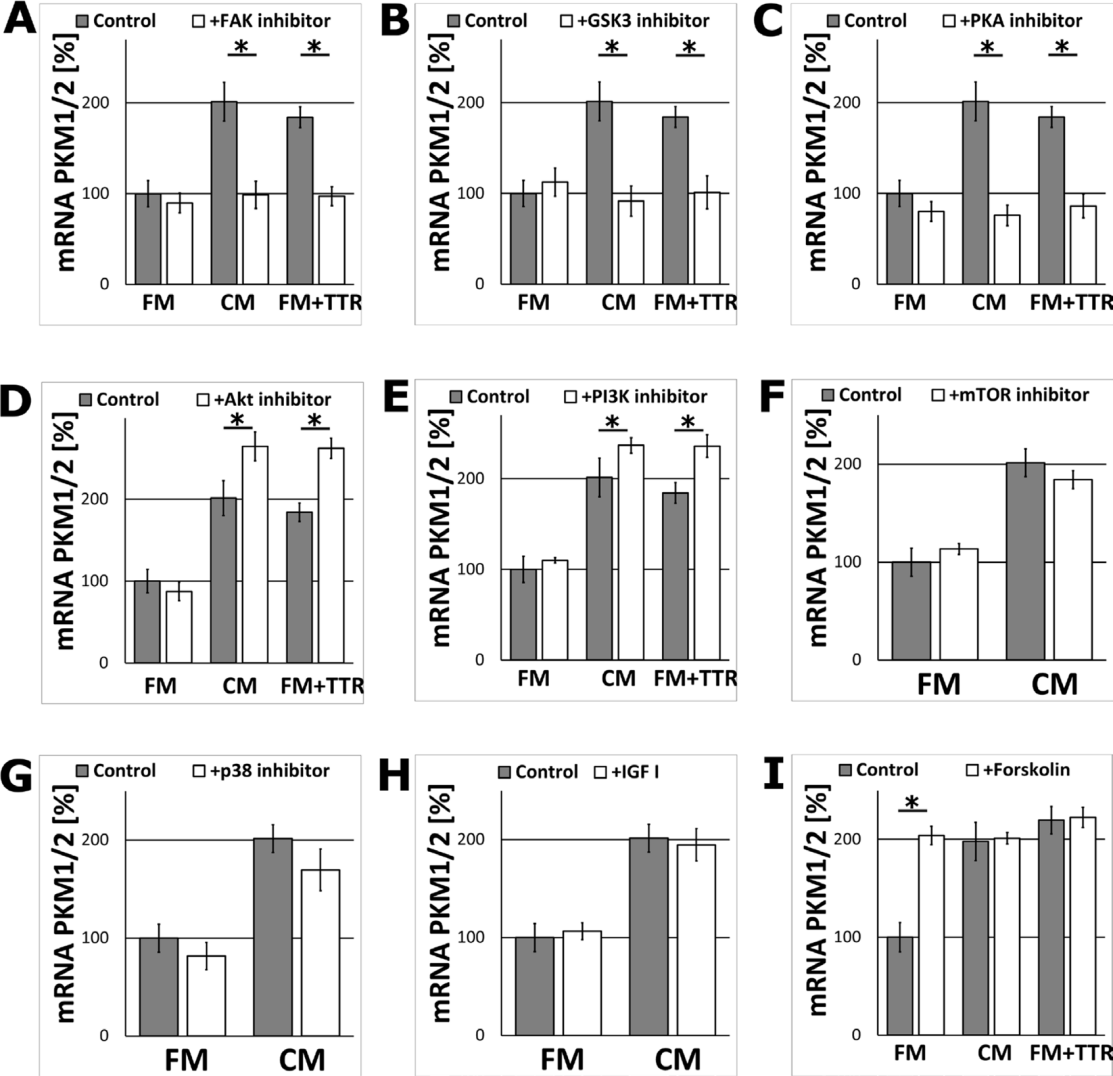
The effect of the metabolic activation or inhibition of the signalling kinases on the expression of the PKM1/2 mRNA in astrocytes The cells were cultured for 24 h in the FM, CM or FM supplemented with 36.4 nM TTR (FM+TTR) media. (**A**–**G**) The effect of inhibition of signalling kinases with (A) 1 μM FAK inhibitor 14, (B) 5 μM SB216763 (GSK3 inhibitor), (C) 20 μM KT5720 (PKA inhibitor), (D) 1 μM AKT inhibitor IV, (E) 2.3 μM wortmannin (PI3K inhibitor), (F) 0.01 μM rapamycin (mTOR inhibitor), (G) 50 μM SB202190 (p38 inhibitor) on the expression of mRNA encoding PKM1/2 in astrocytes. mRNA expression in cells cultured in the FM medium in the absence of the stimulators or inhibitors was assumed to be 100%. Each value represents the mean and S.D. of at least three individual experiments. Asterisks (^*^) indicate statistically significant differences (*P <* 0.001). (**H**–**I**) The effects of the metabolic activators (H) 13 nM IGF-I, (I) 10 μM forskolin on the expression of mRNA encoding PKM1/2 in astrocytes.

Searching for cellular secondary messengers of TTR (and the whole CM) signalling in astrocytes, several activators and inhibitors of proteins kinases crucial for regulation of energy metabolism, were examined. The results demonstrated that inhibition of FAK, GSK3, and PKA in astrocytes treated with TTR or the CM resulted in significant reduction of the level of mRNA encoding PKM1/2 (Figure 6A–6C) compared to the pure CM.

Unexpectedly, the inhibition of AKT and PI3K, the kinases usually involved in the stimulation of energy/glucose metabolism, increased the expression of mRNA encoding PKM1/2 in the CM and TTR-treated cells (Figure 6D, 6E). Inhibition of other studied kinases (mTOR, p38) did not change significantly the level of mRNA encoding PKM1/2 (Figure 6F, 6G). The inhibition of all the above kinases did not affect the expression in the FMtreated cells. Similarly, the incubation of astrocytes with IGF-I did not affect the level of mRNA encoding PKM1/2 (Figure 6H) both, in the FM and the CM-treated cell.

In contrast, supplementation of FM with forskolin (an activator of adenylyl cyclase) imitated the stimulation of the expression observed in the CM. However, the supplementation of the CM with forskolin did not enhance further the increase in PKM1/2 mRNA level (Figure 6I). The similar changes in mRNA level after supplementation of FM with forskolin was observed for PFKP, but not for HK1 (Supplementary Figure 6).

## DISCUSSION

Studies of the brain energetics have revealed that neurons stimulate the expression of glucose metabolism proteins in astrocytes [4]. However, a mechanism of this phenomenon is not well understood [3, 4]. Recently, we have demonstrated that neurons induce genes and proteins expression in astrocytes by releasing of some unknown molecules into extracellular fluid [4]. On the other hand, the study of Hasel *et al.* has shown that a direct cell-to-cell contact also activated the transcription of several metabolic enzymes [5]. Nevertheless, the latter study has not provided any information as to whether the observed transcriptional changes were correlated with altered protein levels and/or the basal metabolic state of astrocytes.

Here, we presented evidence that incubation of astrocytes in the neuron-conditioned medium (CM), but not the fresh medium (FM), increased the astrocytic levels of mRNA and protein and enzymatic activity of pyruvate kinase, a regulatory enzyme of glycolysis.

A significant elevation of the ATP level in the CM-treated astrocytes indicated that neurons stimulate astrocytic energy production by releasing of some molecule(s) into extracellular fluid. Subsequent set of experiments let us not only unequivocally identify transthyretin as the neuron-derived soluble inducer of glycolytic enzymes expression in astrocytes but also demonstrate that the activity of the cAMP-dependent signalling pathway is crucial for these neuron-dependent changes.

TTR was originally described as a carrier protein for T4 and retinol [8, 9]. In the brain, the expression of TTR was thought to be restricted to ependymocytes [10, 23]. However, recent studies have confirmed neuronal synthesis of the molecule [11]. TTR has been shown to play an important role in neuronal plasticity, regeneration and aging [13–17] but its action as a direct metabolic regulator has not been reported thus far. Behavioural deficits observed in TTR knockout mice have suggested that the roles of this protein are more complex than that initially described [13, 24], however, it is noteworthy that some of mentioned defects appear to be abrogated by the administration of retinoic acid [14].

Our studies demonstrated that only the hormone-free TTR acts as an activator of glycolysis in astrocytes and saturation of TTR with T4 or retinol abolished the stimulatory effect of this protein. This finding suggests that TTR is a multifunctional protein and its role in the brain biology is not limited to its action as the carrier protein.

The data presented here point to regulatory enzymes of glycolysis, PKM1/2 and PFKP, as the main targets of TTR action. TTR increased their mRNAs and protein expression level; however, changes in mRNA levels were much faster than those observed for the protein level. The long time lapse between the mRNA and protein increase suggests the existence of an additional step in the regulation of TTR-dependent expression of PK and PFK.

Moreover, the regulation of metabolic enzymes expression by TTR is presumably specific to astrocytes as we did not observed any changes in the expression of PKM1/2 after TTR-treatment in non-glial cells, such as immortalized epithelial cells (ME16C), human fibroblasts, or mouse lung cancer cell line (KLN205).

Taking into account that TTR stimulates the expression of PFK and PKM, but not HK, it may be presumed that TTR acts as a mediator due to which neurons can selectively stimulate oxidation of glucosyl units derived from glycogen but not glucose coming from the blood stream. Thus, the tight coupling of astrocytic and neuronal metabolism may not be necessarily reflected by significant changes of glucose uptake to the glial cells.

For the last twenty years the brain energy metabolism has been analysed in the context of the astrocyte-neuron lactate shuttle (ANLS) hypothesis which assumes that glucose is oxidised in astrocytes to lactate and lactate is used by neurons to fulfil their energy requirements [2, 3]. However, it has also been shown that degradation of glycogen, but not glucose, in astrocytes is a prerequisite for memory formation [25, 26]. The origin of glucosyl units for glycogen synthesis has not been intensively studied but it is commonly believed that they originate from blood-derived glucose. Our present findings lead to conclusion that the higher capacity of astrocytes to degrade glycogen to support neuronal plasticity is not associated with their higher capacity to take up glucose.

A molecular mechanism by which biological information induced by TTR is transmitted within a cell is not fully understood among others, in view of fact that membrane receptor for TTR is thus far unidentified. However, it has been shown that TTR may bind megalin (LRP-2) [27, 28], IGF-IR (insulin-like growth factor 1 receptor) [29] and RAGE (receptor for advanced glycation end products) [30]. Interestingly, it has been demonstrated that megalin-dependent TTR internalization by sensory neurons was necessary for the neuritogenic activity [31] and that TTR-activated megalin stimulated intracellular pathways, such as ERK1/2 and AKT, leading to the activation of transcription factor CREB [32]. The most recent studies have revealed that in hippocampus, TTR promoted neurite outgrowth in a ligand (T4 and retinol)-independent manner via phosphorylation of CREB [33].

The results presented here support the findings that free TTR may activate CREB. We observed that activation of cAMP synthesis and inhibition of PKA, respectively, stimulated and downregulated PKM1/2 level in TTRtreated astrocytes. On the other hand, the inhibition of PI3K/AKT signalling significantly increased PKM1/2 level in TTR-treated cells. Notably, the pharmacological block of GSK3, a target of inhibitory action of AKT (for a review, see [34, 35]) and an activator (synergistically with PKA) of CREB [36, 37], strongly downregulated the expression of PKM1/2. Evidently, the regulation of PKM1/2 in astrocytes by the cAMP/PKA and the PI3K/AKT pathways is antagonistic.

The involvement of cAMP/PKA pathway in the induction of astrocyte-specific PFK and PK expression but not HK elevation is enigmatic. From the functional point of view it makes sense that TTR/cAMP activates only these steps of glycolysis which are engaged in glycogen degradation, not affecting glucose uptake

However, cAMP is a pleiotropic mediator and previous studies have demonstrated that CREB, a cAMP downstream effector, may regulate expression of mammalian HK (hexokinase 2 isozyme) [38]. On the other hand, a newer study has suggested that CREB may be a basic transcription factor for HK expression [39]. Since brain is the organ of the highest basal level of glycolytic capacity [40], an increase in cAMP/PKA activity in astrocytes may not be able to further elevate the expression of HK1, because the level of HK1 already reached its maximum.

Insulin/IGF-I-activation of the PI3K/AKT pathway is associated with acceleration of energy metabolism and is reflected by an increase in glucose uptake [41–43]. Thus, the antagonistic effect of TTR on the cAMP/PKA and the PI3K/AKT pathways is in line with our observation that TTR stimulation of astrocytic metabolism is associated with a activation of glycolytic enzymes which degrade glycogen-derived glucosyl units (PFK, PKM) but not of enzymes stimulating glucose uptake (HK1).

Recent studies have demonstrated that TTR may act via the IGF-IR signalling pathway synergistically with IGFI [44]. However, our results revealed that supplementation with IGF-I did not affect metabolism of neither the FM-treated nor the CM-treated cells. We also did not observe any changes in PKM1/2 level when mTOR or p38 kinases were inhibited. The lack of the effect of IGF-I stimulation on PKM1/2 expression suggests that in astrocytes, the regulation of glycolytic capacity and neurite outgrowth are independently mediated by different TTR-related mechanisms although, supposedly, in the both phenomena, the final decisive role plays the activation of CREB.

**Figure 7:**
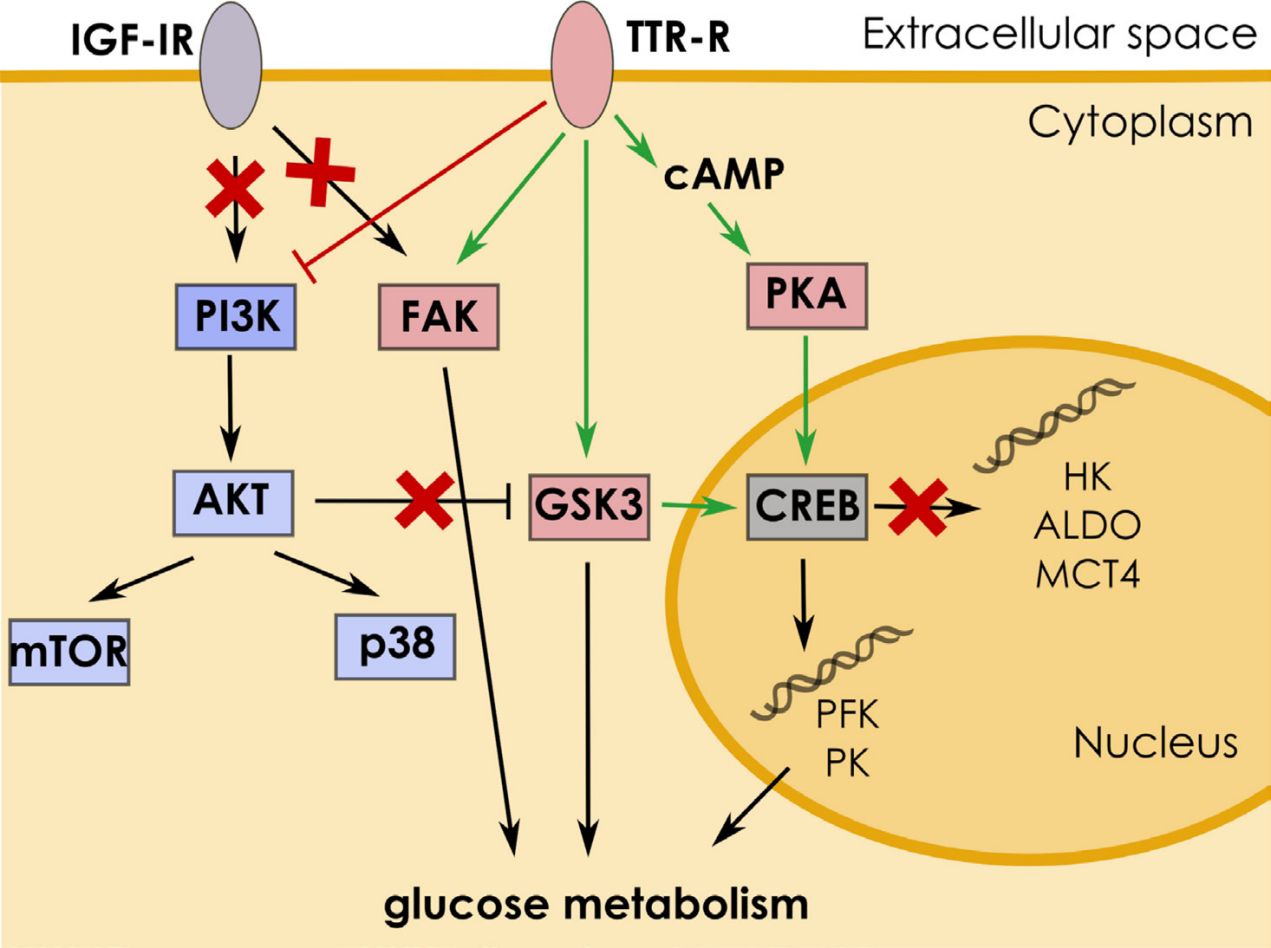
Mechanism of TTR-induced signaling Interaction of TTR with its receptor (TTR-R) triggers signaling cascade leading to stimulation of glucose metabolism in astrocytes. TTR activates CREB via the cAMP/PKA pathway which results in upregulation of PK and PFK expression. The action of TTR is antagonized by the PI3K/AKT pathway, by inhibition of GSK3 activity.

Our studies also revealed that the inhibition of FAK significantly lowered PKM1/2 expression in the CM-treated cells. Just recently, it has been shown that FAK activation by IGF-I was involved in the increase of the expression of several glycolytic genes, among them PKM2, in pancreatic adenocarcinoma [45]. Results presented here excluded the possibility that IGF-I may stimulate expression of PKM1/2 and further studies are required to elucidate the mechanism of FAK-dependent elevation of glycolysis in astrocytes. A diagram depicting a mechanism of TTR induced signalling is presented in Figure 7.

Studying the effect of the CM medium on astrocytic metabolism we also found that the CM, but not TTR, activated the expression of ALDOA. This suggests that not only TTR but also other soluble molecules released from neurons participate in the regulation of astrocytic metabolism. Our preliminary results showed that molecular mass of ALDOA expression activator is below 10 kDa (data not shown). Interestingly, the activation was evident after 24 h incubation of astrocytes with the CM. However, after the next 24 h the FM was also able to stimulate ALDOA expression, which suggests that regulator of ALDOA expression is present also in the fresh neuronal medium, however, its concentration is lower than in the CM.

In conclusion, our results provide strong evidence that TTR is the neuron-derived factor which can specifically stimulate glycolysis in astrocytes in the T4 and retinol-independent manner. The impact of TTR appears to be restricted to the two of three regulatory enzymes of glycolysis, PFK and PKM, which suggests that TTR is involved in the formation of the astrocyte glycogen-neuronal mitochondria metabolic pathway of energy production which is required for memory formation [1, 46].

## MATERIALS AND METHODS

### Cell culture and neuron-conditioned medium preparation

All the procedures were approved by the Local Ethical Commission and every effort was made to minimize the number of animals used for the experiments. All cell cultures were maintained at 37°C in a humidified atmosphere with 5% CO2.

Hippocampal astrocytes and neurones cultures were prepared from newborn Wistar rats and cultured as described previously [4]. To avoid contamination of cultures with neurons and fibroblasts, hippocampal astrocytes were subcultured 2 times [47].

Neurons were cultured for 14–16 days in fresh neuronal medium (FM: Neurobasal-A, 2% B-27 Supplement, 100 U/mL penicillin, 0.1 mg/mL streptomycin, 0.5 mM glutamine, 12.5 μM glutamate, and 25 μM βmercaptoethanol), then the neuron-conditioned medium (CM) was collected, filtered through 0.22 μm filters and stored at –80°C until used.

For *in situ* hybridization and immunofluorescent experiments astrocytes were seeded at a density of 2.0–3.0 10^4^/cm^2^ onto glass coverslips covered with poly-L-lysine (1 mg/mL) and laminin (2.5 μg/mL) and were cultured for 24 h before the experiments. For ATP assay and activity measurement the cells were cultured in their specific medium to 80% confluence.

To check the metabolic changes in astrocytes by neuron-derived molecule(s) [4] astrocytes were incubated for 24 h (or 48 h in some experiments) in the CM. In control experiments, the FM was used.

To get some insight into the chemical nature and the range of the molecular weights of the regulatory factors released by neurons into the culture medium, the CM samples were heated for 5 min at 45°C or 65°C or were filtered through Vivaspin^®^ filters (EQUIMED S.J.). The filtrations resulted in a separation of the CM samples into two fractions: one containing low molecular weight compounds (<30 kDa) and the second, containing both, low and high molecular weight molecules (0–100 kDa).

In order to check the role of TTR and its cargo (retinol, T4) on the expression of metabolic proteins in astrocytes, the FM or the CM medium was supplemented with 20 μg/mL (36.4 nM) of human plasma TTR, 1 μM T4 or 1 μM retinol. In another set of experiments, astrocytes were incubated in the CM depleted of TTR by immunoprecipitation (IP). Briefly, the CM samples were incubated with rabbit anti-TRR antibodies (1:200; Novus Biologicals cat. no. NBP1-50256) overnight at 4°C with gentle agitation, then protein G-coupled Sepharose beads (Roche) were added and incubated overnight at 4°C with agitation. Subsequently, the suspension was centrifuged at 4,000 rpm for 2 min. The supernatant was filtered through 0.22 μm filter and used as the “medium post-IP”. In a control reaction, the primary antibodies were omitted. The results of the controls are shown in (Supplementary Figure 4).

To ascertain whether the effect of the CM and TTR on the expression of PKM1/2 was unique for astrocytes, we examined the expression of PKM1/2 in lung cancer cells (the KLN205 cell line), secondary cultures of human skin fibroblasts and immortalized epithelial cells (the ME16C cell line) seeded in the CM or the FM medium supplemented with TTR. Before the stimulation, the cells were cultured in their specific media up to 80% confluence.

To determine which signalling pathways were engaged in the regulation of the astrocyte energy metabolism, the cells growing for 24 h in the CM or in the FM supplemented with 36.4 nM TTR, were treated for the next 24 h with one of the specific kinase inhibitors: wortmannin (PI3K inhibitor; 2.3 μM), rapamycin (mTOR inhibitor; 0.01 μM), AKT inhibitor IV (AKT inhibitor; 1 μM), FAK inhibitor 14 (FAK inhibitor; 1 μM), SB202190 (p38 inhibitor, 50 μM), SB216763 (GSK3 inhibitor, 5 μM), KT5720 (PKA inhibitor; 20 μM) or with metabolic stimulators: forskolin (adenylyl cyclase activator, 10 μM) or IGF-I (13 nM) and the expression of metabolic proteins were tested.

### Fluorescent *in situ* hybridization (FISH)

5′-end Cy3-labelled oligonucleotides complementary to rat mRNA sequences for PKM1/2 (Cy3AATGG TACAGATGATCCCAGTGTTGCGGGCCGTGATGGG T), PFKP (Cy3-TCAAACACTCCTTT CCCTTCCTCTGA GTAGAGCTGGTAGA) and HK1 (Cy3-AAGTGGGCCA GGGTTTCCTCAATCTGCCGG TGCTGCTCAG) were synthesized by Sigma-Aldrich. FISH was performed according to Mamczur *et al.* [4]. In control reactions, the oligonucleotide probes were omitted. The results of the controls are shown in (Supplementary Figure 1).

### Immunofluorescence

The immunofluorescence studies were performed as described previously [4]. The cells were fixed, permeabilized and incubated with the primary antibodies: goat anti-pyruvate kinase M1/M2 (anti-PKM1/2; 1:1000; Acris Antibodies GmbH cat. no. R1108P), rabbit anti-aldolase A (anti-ALDOA; 1:500; produced and tested as described previously [48, 49]), rabbit anti-phosphofructokinase P (anti-PFKP; 1:200; Novus Biologicals cat. no. NBP1-19585), rabbit anti-hexokinase 1 (anti-HK1; 1:200; Abcam cat. no. AB150423), rabbit anti-monocarboxylate transporter 4 (anti-MCT4; 1:100; Abcam cat. no. AB74109. To analyse a morphology of astrocytes cultured in “neuron-conditioned” and “neuron-unconditioned” media, glial fibrillary acidic protein (GFAP) was visualized with rabbit anti-GFAP antibodies (1:400; Sigma cat. no. G9269). The primary antibodies were detected with fluorophore-labelled secondary antibodies: rabbit anti-goat-FITC (1:1,000; Sigma cat. no. F9012), or goat anti-rabbit-FITC (1:500; Sigma cat. no. F6005). Cell nuclei were stained with DAPI. In controls, the primary antibodies were omitted. The results of the controls are shown in (Supplementary Figure 1).

### Confocal microscopy and fluorescence analysis

The confocal microscopy studies were performed as described previously [4]. The images were taken from 5 to 12 randomly selected areas. All cells observed in the images (about 10 cells per image) were used to measure the mean fluorescence. The quantification of the fluorescence was performed using Cell^F software (Olympus Soft Imaging Solutions GmbH). For analysis of mRNA and proteins expression, the edges of the cells were marked and the mean fluorescence was measured. All experiments were done in triplicate.

### Activity measurement

PKM1/2 activity was assayed spectrophotometrically as described previously [40], with slight modifications. Enzyme activity was assayed using cellular extract obtained by homogenisation of astrocytes in a lysis buffer (250 nM KCl, 50 mM Tris, 0.1% Triton x-100, 0.1 mM EDTA, 0.1 mM EGTA, pH 7.4, 4°C). To avoid degradation of cytosolic proteins protease inhibitor cocktail (1:25, Roche) was added. Thereafter, the homogenate was centrifuged at 20,000 g, at 4°C, for 20 min. The activity was measured in 50 mM Bis-Tris-Propane buffer with 100 mM KCl, 0.25 mM EDTA and 10.25 mM MgCl2, pH 7.4, 37°C. The reaction mixture contained 0.2 mM NADH, 1.25 mM ADP and 1 U LDH. An enzyme activity, expressed in U [μmol min−1] was determined from the difference in the slope of NADH absorbance (340 nm; ε = 6.22 mM−1 cm−1) after addition of a substrate (5 mM phosphoenolpyruvate). All spectrophotometric measurements were performed with an Agilent 8453 diode array spectrophotometer. Protein concentrations were determined using Bradford Reagent. All measurements were done in triplicate.

### Mass spectrometry

Media were filtered as described above. Obtained supernatant (fraction 30–100 kDa) was precipitated with TCA, resolved by 15 % SDS-PAGE and stained with PageBlue™ Protein Staining Solution (Thermo Fisher Scientistic). Acrylamide gels were then cut into 0.5 cm slices. In this way we obtained 16 slices (8 per each medium) containing separated proteins.

Protein within slices were reduced with DTT, alkylated with iodoacetamide and digested with trypsin according to the standard protocol [50]. A total of 40 ng of sequencing grade trypsin was added to each excised gel fragment, covered with 100 mM ammonium bicarbonate and the samples were incubated at 37°C overnight. The supernatant was collected and further extraction was performed by addition of 5% formic acid/ACN (1:1, v/v) followed by a second extraction step of 5% formic acid/ACN (5:95, v/v). The combined solvent extracts were vacuum dried and reconstituted in 0.1% TFA, concentrated and desalted using C_18_ ZipTips (Millipore). The obtained peptides were analysed by MALDI-TOF/TOF using the Applied Biosystems 4800 Proteomics Analyser. Sample preparation for analysis was as follows: tryptic peptides were mixed 1:1 v/v with α-cyano-4-hydroxycinnamic acid (10 mg/mL in ACN/0.1% TFA) and spotted directly onto the MALDI target without chromatographic separation. MS spectra were calibrated using two external standards and tryptic autolytic peaks. Typically, each MS spectrum was acquired in the positive-ion reflectron mode using 400 laser shots. A set of 6–20 ions, depending on the complexity of the MS1 spectrum, which had both (1) strong intensities and (2) were well resolved from neighbouring ions were analysed further. Fragmentation spectra were acquired as postsource decay unimolecular decompositions (collision gas off) using 3,000 laser shots. A fragmentation voltage of 2 kV was used throughout the automated runs.

The spectra were processed and analysed using the 4000 Series Explorer v3.5.3 (Applied Biosystems) and ProteinPilot v4.0 (Applied Biosystems). Protein searches were performed against the SwissProt Homo sapiens protein database (release date 06032017) using Mascot 2.3 and Paragon 4.0 platform. The database search parameters were: mass tolerance of 40 ppm for precursor ions and 0.8 Da for fragment ions; trypsin digestion with two missed cleavages, fixed modification–carbamidomethylation (C). Protein identity was accepted at the 95% confidence level.

### ATP measurement

ATP measurements were performed with Single Tube Luminometer (Microdigital) according to the manufacturer requirements. ATP level was determined in a cellular extract obtained by incubation of the cells in an extraction buffer (50 mM Tris, 1 mM EDTA, 0.1% Triton X-100, pH 7.5, RT) for 10 min. The lysates were heated for 45s at 99°C and then centrifuged at 10,000 g at 4°C for 10 min. Then, the supernatants were collected and assayed with luciferase/luciferin (Sigma) according to manufacturer recommendations. Protein concentrations were determined using Bradford Reagent. All measurements were performed at least in triplicate.

### Western blot

Protein extracts were obtained by incubation of astrocytes in a lysis buffer (50 mM Tris, 0.2 mM EDTA, 5% SDS, 50 mM DTT, pH 8.0) for 20 min at 99°C and centrifuged at 20,000g at 4°C for 20 min. The supernatants were collected and total protein concentration was determined using the Bradford method. The extracts (15 μg) were resolved by 10% SDS-PAGE, transferred to the nitrocellulose using a wet system and stained with Poceau S to test the quality of the protein transfer. Membrane was blocked for 1 h in 3% BSA in PBS and then incubated overnight at 4°C with primary antibodies diluted in Solution 1 from SignalBoost™ Immunoreaction Enhancer Kit (Millipore). Then the blot was incubated for 2 h at RT with secondary antibodies diluted in Solution 2 from SignalBoost™ Immunoreaction Enhancer Kit. Pyruvate kinase M1/2 was detected with goat anti-PKM1/2 antibodies (1:2500; Acris Antibodies GmbH cat. no. R1108P), secondary antibodies conjugated with biotin (1:2500; Sigma cat. no. B7014) and Extravidin conjugated with peroxidase (1:2000 in PBS; Sigma cat. no. E2886). Mouse anti-β-actin antibodies (1:2500; Sigma cat. no. A5441) and secondary antibodies conjugated with peroxidase (1:2500; Sigma cat. no. A4416) were used to detect β-actin which was used as a loading control. A peroxidase substrate, 3,3′-diaminobenzidine (DAB), was used.

### Statistical analysis

Collected data was analysed using Microsoft Excel 2010. Results are expressed as mean and standard deviation of at least three individual experiments. For an evaluation of statistical significance the Student’s *t*-test was used. A probability of *P* < 0.05 was considered to represent a significant difference.

## SUPPLEMENTARY MATERIALS FIGURES AND TABLE



## Abbreviations

AKT: protein kinase B
ALDOA: aldolase A
ANLS: astrocyte-neuron lactate shuttle
CM: neuron-conditioned medium
CREB: cAMP response element-binding protein
FAK: focal adhesion kinase
FM: fresh neuronal medium
GSK3: glycogen synthase kinase 3
HK1: hexokinase 1
IGF I: insulin-like growth factor 1
MCT4: monocarboxylate transporter 4
mTOR: mechanistic target of rapamycin
p38: p38 mitogen-activated protein kinase
PFK: phosphofructokinase
PI3K: phosphatidylinositol-3-kinase
PKA: protein kinase A
PKM: pyruvate kinase M
T4: thyroxine
TTR: transthyretin
