# Supplementary material for: Neuron-derived transthyretin modulates astrocytic glycolysis in hormone-independent manner
Source: Oncotarget. 2017 Nov 20;8(63):106625–38. doi: 10.18632/oncotarget.22542 (PMC5739761; doi:10.18632/oncotarget.22542)
Supplement: Supplementary file 1 [file oncotarget-08-106625-s001.pdf]

Neuron-derived transthyretin modulates astrocytic glycolysis in hormone-independent manner

SUPPLEMENTARY MATERIALS

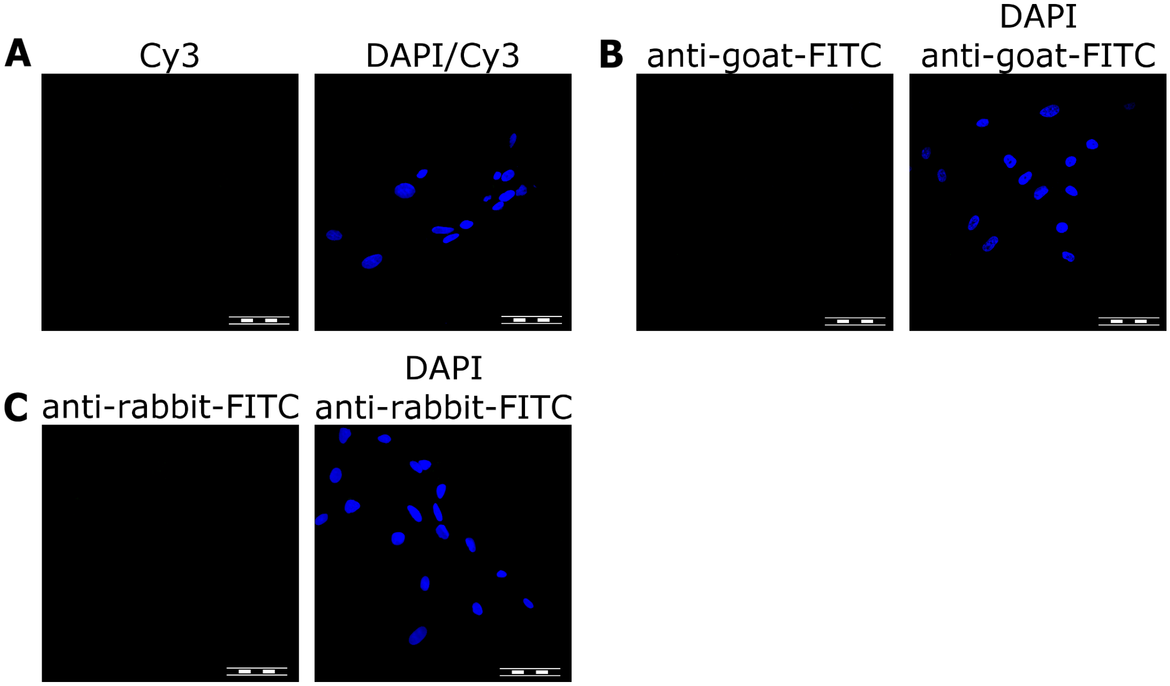

Supplementary Figure 1: Negative controls for FISH and immunofluorescent experiments. In the controls for FISH experiments, the antisense Cy3-labeled oligonucleotides were omitted (**A**) and in the immunofluorescent experiments, the primary antibodies were omitted (**B**, **C**). The results demonstrated the absence of fluorophore-related fluorescence. The nuclei were visualized with DAPI. Bar = 50 µm.

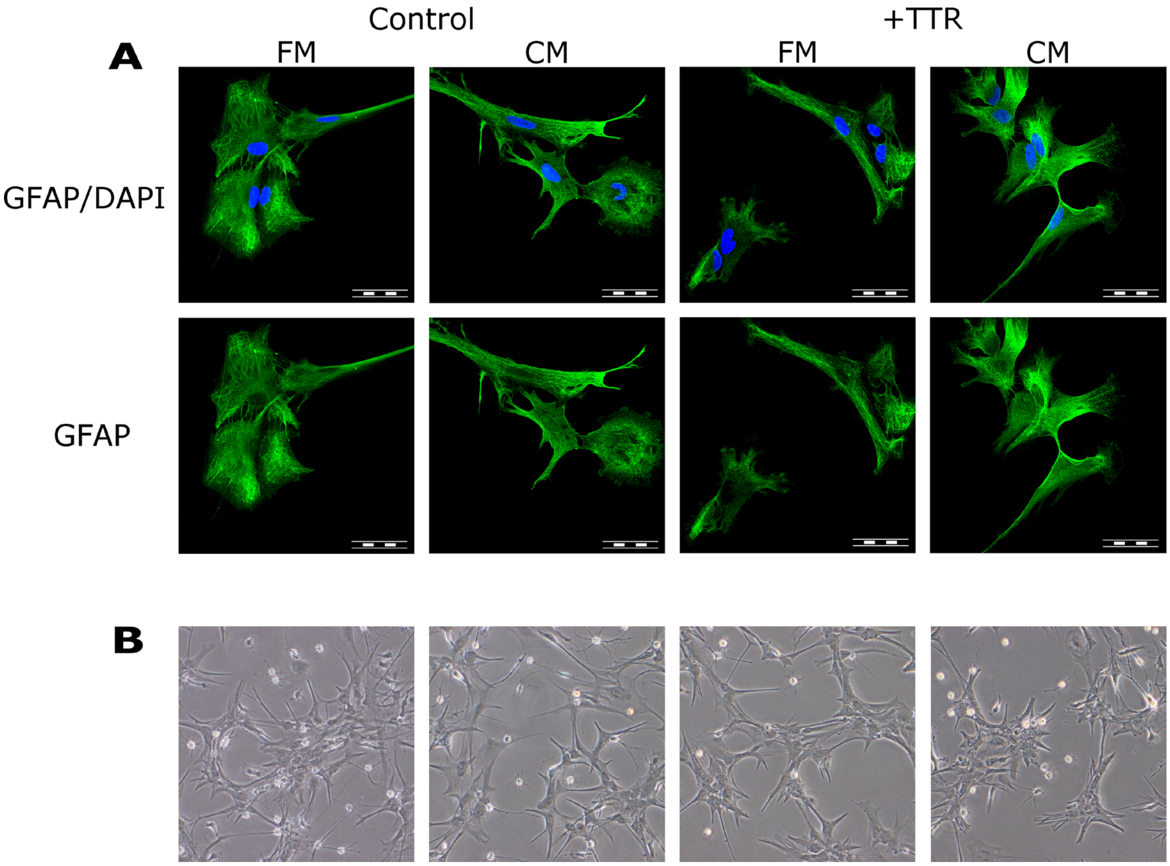

Supplementary Figure 2: The effect of the CM and TTR on astrocytes morphology. (**A**) Subcellular localization of GFAP in astrocytes cultured for 48 h in the FM or the CM supplemented with 36.4 nM TTR (+TTR). The nuclei were visualized with DAPI. Bar = 50 µm. (**B**) Phase-contrast microscopy of astrocytes cultured for 24 h in the FM or the CM supplemented with 36.4 nM TTR (+TTR).

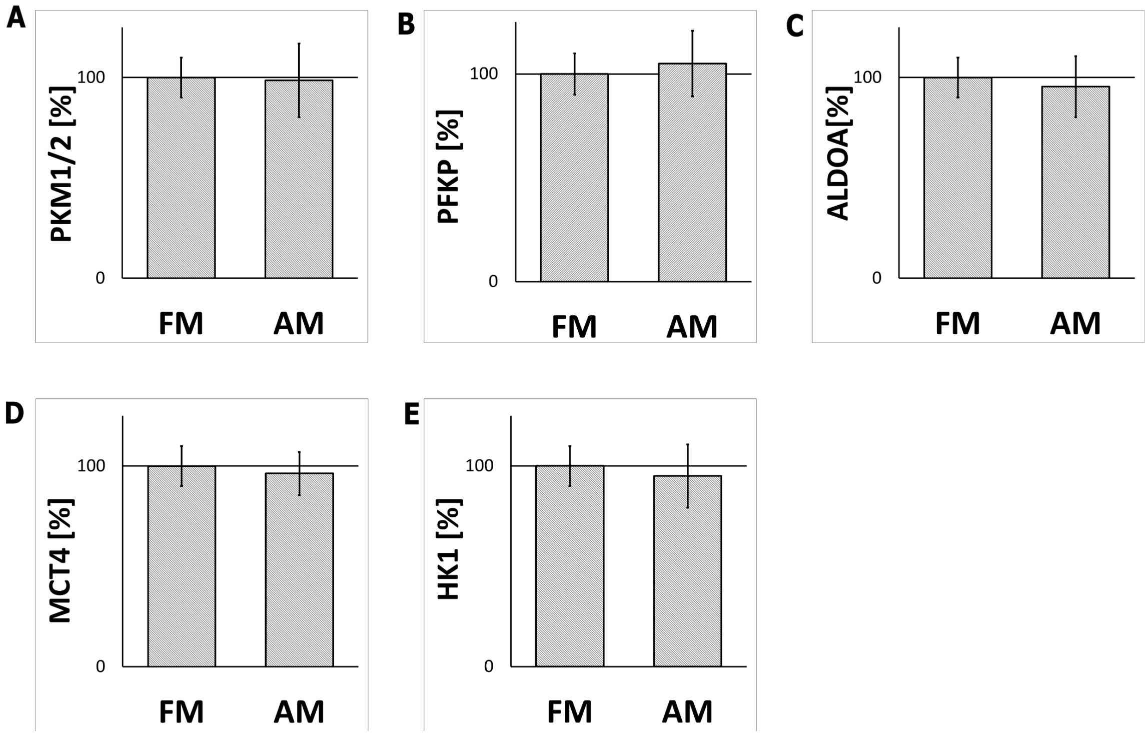

Supplementary Figure 3: The effect of the astrocytic medium (AM) and the fresh neuronal medium (FM) on ALDOA, HK1, MCT4, PFKP and PKM1/2 expression in astrocytes. The measurement of PKM1/2 (**A**), PFKP (**B**), ALDOA (**C**), MCT4 (**D**), HK1 (**E**) protein-related immunofluorescence in astrocytes cultured for 48 h in the astrocytic medium and the fresh neuronal medium revealed lack of medium-dependent changes in expression of the enzymes. The expression of the enzyme in cells cultured in the FM medium was assumed to be 100%. Each value represents the mean and S.D. of at least three individual experiments.

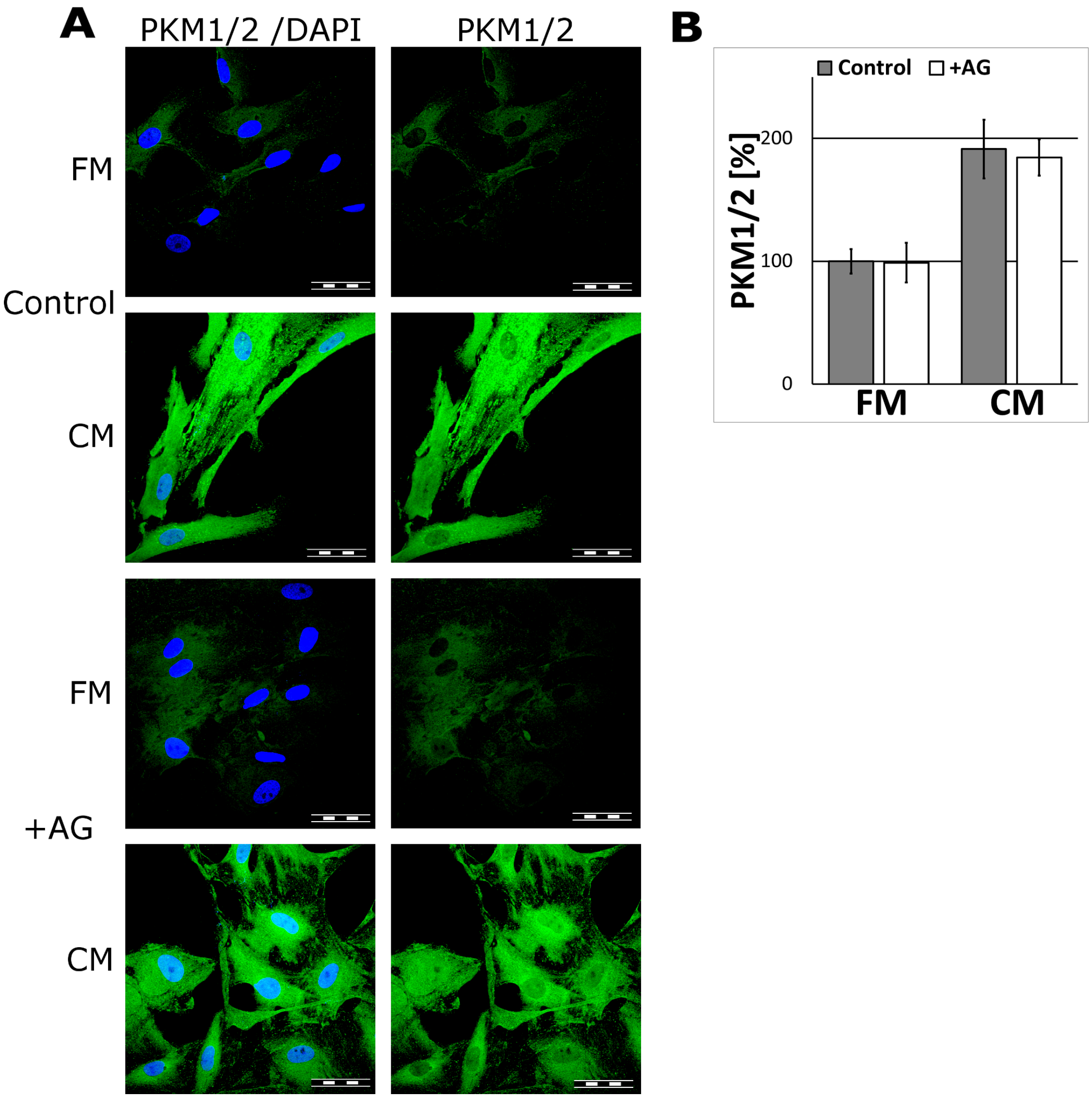

Supplementary Figure 4: Controls for IP experiment. (**A**) Subcellular localization of PKM1/2 in astrocytes cultured for 48 h in the FM and the CM (control stainings) and in the FM and the CM media treated with agarose G (+AG) but with omission of anti-TTR antibodies. (**B**) The counting of PKM1/2 protein-related immunofluorescence in astrocytes cultured as it is described above (in A). The expression of the enzyme in cells cultured in the FM medium was assumed to be 100%. Each value represents the mean and S.D. of at least three individual experiments. The nuclei were visualized with DAPI. Bar = 50 µm.

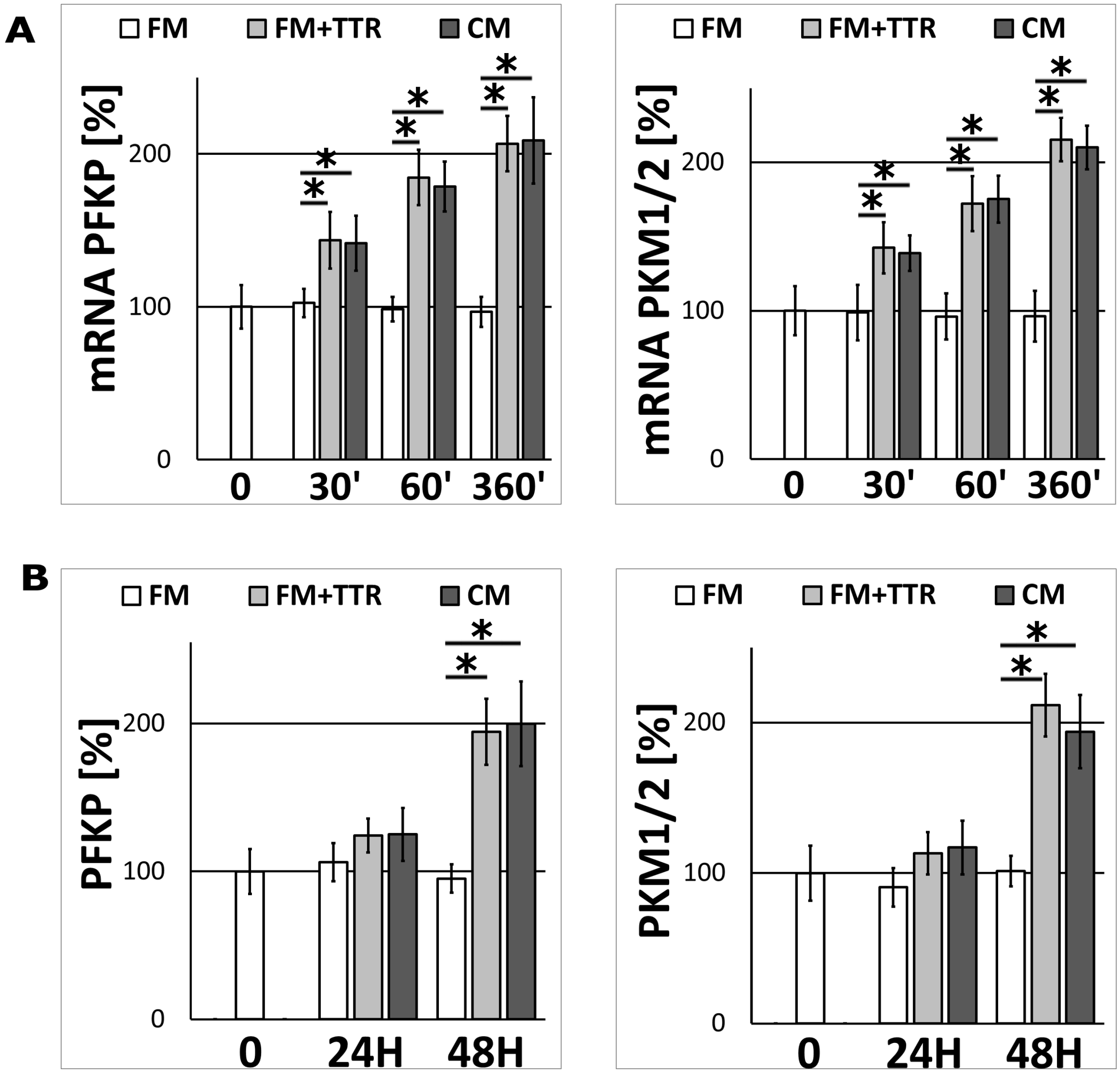

Supplementary Figure 5: The time-dependent changes of PFKP and PKM1/2 expression in astrocytes. (**A**) The effect of culturing for 30, 60 or 360 min in the FM, CM and FM supplemented with 36.4 nM TTR (FM+TTR) media on the level of mRNA encoding PFKP (left chart) or PKM1/2 (right chart) in astrocytes. (**B**) The counting of PFKP (left chart) or PKM1/2 (right chart) protein-related immunofluorescence in astrocytes cultured for 24 or 48 h in the FM, CM and FM supplemented with 36.4 nM TTR (FM+TTR). The expression of the enzymes in cells cultured in the astrocytic medium (0) was assumed to be 100%. Each value represents the mean and S.D. of at least three individual experiments. Asterisks (*) indicate statistically significant differences ($P < 0.001$).

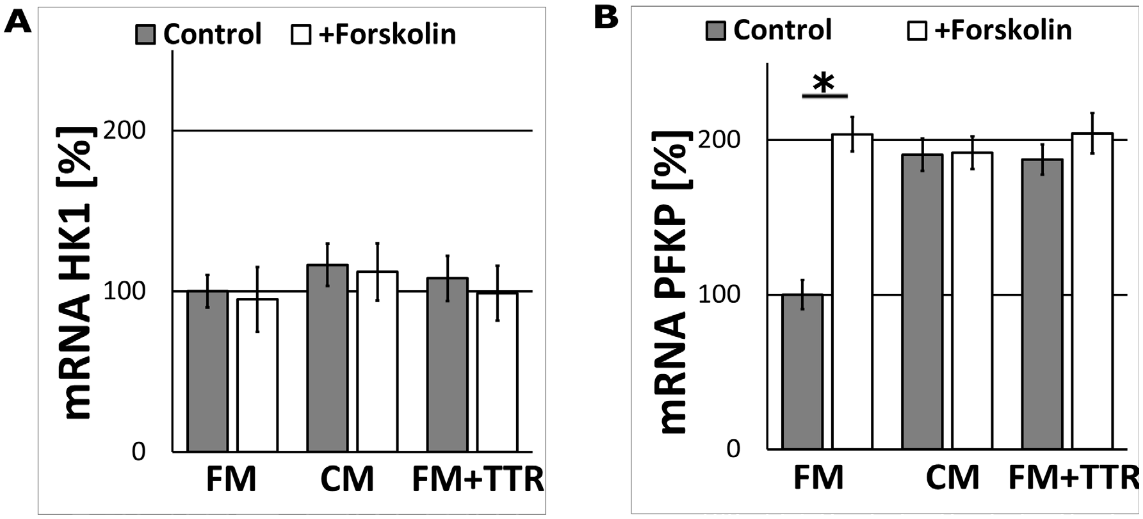

Supplementary Figure 6: The effect of forskolin on the level of mRNA encoding HK1 and PFKP in astrocytes. (**A–B**) The effect of 10 μM forskolin on the level of mRNA encoding HK1 (A) and PFKP (B) in astrocytes. mRNA expression in cells cultured in the FM medium in the absence of the forskolin was assumed to be 100%. Each value represents the mean and S.D. of at least three individual experiments. Asterisk (*) indicates statistically significant differences ($P < 0.001$).

Supplementary Table 1: Proteins identified in fractions 30–100 kDa of the FM and CM media using MS analysis

Sample	% cov	Name	Species	Peptides (95%)
FM1	15.3	Q9BTL3	RAM_HUMAN	RNMT-activating mini protein FAM103A1
	5.7	Q9H293	IL25_HUMAN	Interleukin-25 IL25
	5.4	P62834	RAP1A_HUMAN	REVERSED Ras-related protein Rap-1A RAP1A
	3.7	Q9BS16	CENPK_ HUMAN	Centromere protein K CENPK
FM2	8.5	B7Z368	CJ142_HUMAN	Uncharacterized protein C10orf142 C10orf142
	6.7	Q01469	FABP5_HUMAN	Fatty acid-binding protein, epidermal FABP5
FM3	6.7	Q96IX9	A26L1_HUMAN	Putative ankyrin repeat domain-containing protein 26-like 1 ANKRD36BP1
FM4	3.9	P61599	NAA20_HUMAN	N-alpha-acetyltransferase 20 NAA20
FM5	3.9	P61599	NAA20_HUMAN	N-alpha-acetyltransferase 20 NAA20
FM6	3.9	P61599	NAA20_HUMAN	N-alpha-acetyltransferase 20 NAA20
	3.6	P02768	ALBU_HUMAN	Serum albumin ALB
FM7				NO IDENTIFIED PROTEINS
FM8				NO IDENTIFIED PROTEINS
CM1	15.7	P02766	TTHY_HUMAN	Transthyretin TTR
CM2	5.4	P62834	RAP1A_HUMAN	REVERSED Ras-related protein Rap-1A RAP1A
CM3				NO IDENTIFIED PROTEINS
CM4	3.9	P61599	NAA20_HUMAN	N-alpha-acetyltransferase 20 NAA20
CM5				NO IDENTIFIED PROTEINS
CM6	3.6	P02768	ALBU_HUMAN	Serum albumin ALB
CM7	4.6	P02768	ALBU_HUMAN	Serum albumin ALB
CM8	3.6	Q96DC8	ECHD3_HUMAN	REVERSED Enoyl-CoA hydratase domain-containing protein 3, mitochondrial ECHDC3

Acrylamide gels (containing separated proteins from fraction 30–100 kDa of the FM and CM media) were cut into 0.5 cm slices. In this way 16 slices (8 per each medium) were obtained. Peptides extracted from gel slices were analyzed by MALDI-TOF/TOF. Proteins are recorded as a solid identification only for hits with % coverage >3.5.

Sequence differences were not sufficient to distinguish between rodent, human and bovine sequences. For this reason all hits refer to human sequences. Some proteins exist in multiple forms, with different molecular weights and isoelectric points. For example, N-alpha1-acetyltransferase 20 was found in three different locations in CM. At present, it is not clear if this heterogeneity is due to proteolysis, secondary modifications, or splice variation. While it is unlikely that the extracellular proteins assayed in cultured medium are derived from cell lysis [1], we cannot rule out the possibility that some lysis occurs that is detected by the highly sensitive LC/MS technology, especially in the face of detection of mitochondrial protein.

Among the detected proteins, three were presented both in conditioned and un-conditioned medium suggesting, that they are components of pure neuronal medium. Interestingly, six proteins were found only in the FM.

1. Schubert D, Herrera F, Cumming R, Read J, Low W, Maher P, Fischer WH. "Neural cells secrete a unique repertoire of proteins." J Neurochem. 2009; 427–435.
